# Birbeck Granule-Like “Organized Smooth Endoplasmic Reticulum” Resulting from the Expression of a Cytoplasmic YFP-Tagged Langerin

**DOI:** 10.1371/journal.pone.0060813

**Published:** 2013-04-05

**Authors:** Cédric Lenormand, Coralie Spiegelhalter, Bertrand Cinquin, Sabine Bardin, Huguette Bausinger, Catherine Angénieux, Anita Eckly, Fabienne Proamer, David Wall, Ben Lich, Sylvie Tourne, Daniel Hanau, Yannick Schwab, Jean Salamero, Henri de la Salle

**Affiliations:** 1 Unité Mixte de Recherche Santé 725, Institut National de la Santé et de la Recherche Médicale, Strasbourg, France; 2 Université de Strasbourg, Strasbourg, France; 3 Histocompatibility Laboratory, Etablissement Français du Sang-Alsace, Strasbourg, France; 4 Imaging Centre, Institut de Génétique et de Biologie Moléculaire et Cellulaire, Illkirch, France; 5 Molecular Mechanisms of Intracellular Transport Laboratory, Unité Mixte de Recherche 144 Centre National de la Recherche Scientifique-Institut Curie, Paris, France; 6 Cell and Tissue Imaging Facility, BioImaging Cell-Institut Curie and Tissue Core Facility & Nikon Imaging Center, Unité Mixte de Recherche 144 Centre National de la Recherche Scientifique-Institut Curie, Paris, France; 7 Soleil Synchrotron, Gif-sur-Yvette, France; 8 Unité Mixte de Recherche Santé 949, Institut National de la Santé et de la Recherche Médicale, Strasbourg, France; 9 FEI Company, Eindhoven, The Netherlands; The University of Queensland, Australia

## Abstract

Langerin is required for the biogenesis of Birbeck granules (BGs), the characteristic organelles of Langerhans cells. We previously used a Langerin-YFP fusion protein having a C-terminal luminal YFP tag to dynamically decipher the molecular and cellular processes which accompany the traffic of Langerin. In order to elucidate the interactions of Langerin with its trafficking effectors and their structural impact on the biogenesis of BGs, we generated a YFP-Langerin chimera with an N-terminal, cytosolic YFP tag. This latter fusion protein induced the formation of YFP-positive large puncta. Live cell imaging coupled to a fluorescence recovery after photobleaching approach showed that this coalescence of proteins in newly formed compartments was static. In contrast, the YFP-positive structures present in the pericentriolar region of cells expressing Langerin-YFP chimera, displayed fluorescent recovery characteristics compatible with active membrane exchanges. Using correlative light-electron microscopy we showed that the coalescent structures represented highly organized stacks of membranes with a pentalaminar architecture typical of BGs. Continuities between these organelles and the rough endoplasmic reticulum allowed us to identify the stacks of membranes as a form of “Organized Smooth Endoplasmic Reticulum” (OSER), with distinct molecular and physiological properties. The involvement of homotypic interactions between cytoplasmic YFP molecules was demonstrated using an A206K variant of YFP, which restored most of the Langerin traffic and BG characteristics observed in Langerhans cells. Mutation of the carbohydrate recognition domain also blocked the formation of OSER. Hence, a “double-lock” mechanism governs the behavior of YFP-Langerin, where asymmetric homodimerization of the YFP tag and homotypic interactions between the lectin domains of Langerin molecules participate in its retention and the subsequent formation of BG-like OSER. These observations confirm that BG-like structures appear wherever Langerin accumulates and confirm that membrane trafficking effectors dictate their physiology and, illustrate the importance of molecular interactions in the architecture of intracellular membranes.

## Introduction

The use of fluorescent proteins (FPs) has revolutionized cell biology. Following the initial purification of Green Fluorescent Protein (GFP) from the jellyfish *Aequorea victoria* [1], the cloning of the GFP gene [2] has led to a continuously expanding list of applications, through the development of a variety of improved GFP variants. In particular, the use of genetically encoded optical tags allows one to visualize the proteins of interest in live cells, opening up fascinating possibilities. However, caution is required when engineering a new FP fusion protein, in order to avoid potentially misleading artifacts [3]. Among the classical sources of such aberrant results, the tendency of many FPs [including enhanced (E)GFP and yellow (Y)FP] to spontaneously oligomerize is well known and can lead to undesirable interactions between FP fusion proteins [4].

In cultured cells, the ER is a highly dynamic subcellular compartment which forms an interconnected network of tubular and laminar structures, either ribosome-covered (rough ER) or ribosome-free (smooth ER). Changes in the architecture of the ER can be induced by different signals, leading to the accumulation of membranes in various highly ordered structures (e.g., stacked cisternae called karmellae, concentric membrane whorls, packed undulating sinusoidal ER, crystalloid ER) which have been grouped under the term OSER [5]. OSER can be induced by the over-expression of particular ER resident transmembrane proteins such as cytochrome b5 [5], HMG-CoA reductase [6], TMPO [7], calnexin [8], VAPB [9]. One may note that the fusion of a GFP tag to the cytoplasmic domain of a resident ER protein can be sufficient to trigger the formation of typical OSER [5]. The formation of OSERs is largely dependent on the ability of ER resident membrane proteins to form multimers via their cytoplasmic tail [10], so the ability of GFP tag to form dimers explains its ability to induce OSER [4], [5]. Topologically, OSER induced by protein hyperexpression can be related to cubic membranes, which are highly convoluted membrane organizations displaying symmetries and 3-dimensional periodicities present in many biological systems [11], [12].

Langerin (CD207), a type II transmembrane cell surface receptor with a C-type lectin-like domain, is expressed in Langerhans cells, the resident immature dendritic cells of the human epidermis [13]. This protein allows the capture and the internalization of antigens or viruses and their subsequent presentation by CD1a or histocompatibility class II molecules [14], [15].

Langerin is naturally present on the plasma membrane and is constitutively internalized, thereby gaining access to the sorting endosomes before being routed to the tubulo-vesicular network of the endosomal recycling compartment. In these structures, Langerin accumulates in specific subdomains, the so-called “Birbeck granules” (BGs) [16]. These subcellular organelles appear as rod-shaped pentalamellar structures of different sizes, with a distinctive central striated lamella [17]. Once a critical molecular concentration has been reached, the carbohydrate-recognition domain (CRD) of Langerin allows zipping of the membranes through homotypic interactions, i.e., the formation of BGs [18]. Finally, Langerin recycles to the plasma membrane [16], [19]. As a subcellular organelle, BGs thus constitute a paradigm of dynamic cellular architecture and potentially illustrate the ultrastructural consequences of the coordinated kinetics of membrane traffic.

To study the dynamics of Langerin traffic, a Langerin-YFP fusion protein (Lang-YFP, luminal or extracellular YFP tag) was expressed in the M10 human melanoma cell line [19]. Fluorescent live cell imaging allowed the observation and quantification of numerous transient fluorescence concentration events in the pericentriolar region of the cells, where BGs are known to accumulate, and close to or at the plasma membrane. These observations depicted dynamic processes involving the endocytosis, sorting and exocytosis of Langerin-positive vesicles. The relevant cellular transport steps were found to be strictly controlled by the formation of a myosin Vb/Rab11A/Rab11-FIP2 platform, firstly in endosomal recycling compartments, where Langerin-positive vesicles are formed and sorted, and secondly at the very late stage of docking/tethering and fusion of these vesicles with the plasma membrane [20].

However, none of these previous studies permitted the direct appreciation of an eventual relationship between the complex dynamics and the ultrastructural characteristics of BGs in cells. In this paper, we therefore investigated whether a cytoplasmic YFP-Langerin fusion protein (YFP-Lang) could likewise be used to model the impact of the cellular transport of a cargo protein on organelle biogenesis and in particular the effect of Langerin on BGs. Our observations extend beyond the framework of the Langerin/BG context and also have technological implications, as well as consequences for the “in and out” of transmembrane proteins in cellular membranes.

## Materials and Methods

### Antibodies

The mouse anti-Langerin mAb DCGM4 was purchased from Dendritics (Lyon, France), rabbit anti-calnexin (SPA865) Abs from Stressgen and, rabbit anti-BiP (Ab32618) Abs from Abcam (Paris, France). HRP-conjugated goat anti-GFP Abs were from Miltenyi Biotec (Paris, France) and secondary Cy5-conjugated donkey anti-rabbit Abs from Jackson ImmunoResearch (West Grove, PA).

### Cell culture and transfection

The M10 human melanoma cell line [21] and its transfected M10-22E [22] and M10-Lang-YFP [19] derivatives have been previously described. To express a Langerin protein fused to YFP at its N-terminal cytoplasmic end (YFP-Lang) in M10 cells, the CD207 cDNA was cloned in the plasmid pEYFP-C3 (Clontech, Ozyme, Paris, France). The monomerizing A206K mutation was introduced into the YFP sequence by PCR (mYFP-Lang) and both constructions were verified by DNA sequencing. The LangE293A variant was obtained by PCR techniques.

Stable M10-YFP-Lang and M10-mYFP-Lang cell lines were obtained by transfection of M10 cells using Fugene 6 reagent (Roche Applied Science, Meylan, France) followed by selection of the clones with 400 µg/mL G418 (Invitrogen Fischer Scientific, Illkirch-Graffenstaden, France). All cells were grown in RPMI 1640 supplemented with 10% heat-inactivated fetal calf serum, penicillin and streptomycin (Invitrogen Fischer Scientific).

### Immunofluorescence confocal microscopy

Adherent cells were cultured overnight on a 12 mm diameter glass coverslip. The cells werefixed in cold methanol and treated with 0.1% Triton ×100 and according to standard procedures. Preparations were mounted in Mowiol (Citifluor, Biovalley, Marne-la-Vallée, France) and images were obtained with the HCX PL APO lambda blue 63.0×1.40 objective of a Leica SP5-AOBS confocal microscope (Leica Microsystems, Heidelberg, Germany). For confocal microscopy, eYFP was excited with at 488 nm, emission was captured using a 515–560 nm AOBS filter. Cy5 fluorophore was excited using at 633 nm, emission was captured using a 650–730 nm AOBS filter.

### Transmission electron microscopy (TEM), immunogold electron microscopy, correlative light-electron microscopy (CLEM) and focused ion beam/scanning electron microscopy (FIB/SEM)

For TEM, cells were first fixed in 0.1 M sodium cacodylate buffer containing 2.5% glutaraldehyde, postfixed in 1% osmium tetroxide and en-bloc stained with 2% uranyl acetate. The samples were then dehydrated in graded ethanol solutions and embedded in Epon (Ladd Research Industries, Inland Europe, Conflans sur Lanterne, France). Ultrathin sections (100 nm) were examined under a Philips CM120 BioTwin (120 kV) electron microscope (FEI Company, Eindhoven, The Netherlands).

For immunogold electron microscopy, cells were fixed with 2% paraformaldehyde and 0.2% glutaraldehyde in 0.1 M phosphate buffer. The cell pellets were then infiltrated in 2.3 M sucrose and frozen in liquid nitrogen and the samples were cut into 70 nm-thick cryosections. The sections were incubated with 10 µg/mL polyclonal rabbit anti-GFP antibodies (Invitrogen) or mouse anti-Langerin (DCGM4; Beckman-Coulter), anti-KDEL peptide (10C3; Calbiochem) or anti-protein disulfide isomerase (1D3; Enzo) mAbs. Rabbit anti-mouse IgGs were used as bridging Abs. The sections were fixed, counterstained with protein A-conjugated 10 nm gold beads and viewed under a CM120 electron microscope (80 kV).

For CLEM, adherent cells were first cultured on laser micro-patterned Aclar® supports [23]. Cells of interest were selected, precisely located and imaged by fluorescence confocal microscopy using a Leica TCS SP2-AOBS microscope. The samples were then processed exactly as for TEM and ultrathin sections (50–60 nm) were examined under a Philips CM12 electron microscope (80 kV) equipped with an Orius 1000 ccd camera (Gatan, Roper Scientific, Evry, France).

For FIB/SEM, cells were also processed as for TEM except that a contrast-enhancing step, consisting of incubating the cells in 1.5% potassium ferrocyanide and 1% osmium tetroxide in 0.1 M sodium cacodylate buffer, was added directly after the fixation step. The blocks were mounted on SEM stubs, coated with platinum/palladium and examined under a Helios NanoLab dual beam microscope (FEI Company). Samples were milled at a thickness of 20 nm per slice using the FIB (30 kV, 700 pA) and imaged with a backscattered imaging mode (3 kV, 1 nA).

Tomograms and 3D models were computed using IMOD software (http://bio3d.colorado.edu/imod/) [24].

### Fluorescence recovery after photobleaching (FRAP)

In FRAP experiments, time series were acquired using a spinning disk microscope (Roper Scientific) based on a CSU-X1 Yokogawa head mounted on the lateral port of an inverted Ti-Eclipse Nikon microscope equipped with a 100× 1.4NA Plan-Apo objective and a fibered 491 nm 50 mW DPSS laser (Roper Scientific). Images were obtained with a Photometrics Coolsnap HQ2 CCD camera (Photometrics, Tucson, AZ). Bleaching was performed using a combined FRAP-4D module at the back illumination port of the microscope, previously developed by some of us and commercialized under the name of *Ilas1* (Roper Scientific). All the imaging modalities of this system were controlled with Metamorph 7.1.7 software (MDS Analytical Technologies, Sunnyvale, CA). Stacks of images (4 to 6 planes) were taken approximately every 1.6 s. Following the first 5 to 12 images, a selected region of interest (ROI) was bleached at about 60 to 80% acousto-optic tunable filters-controlled laser power in ∼75 ms. The recovery of the fluorescent signal within the ROI and the total fluorescence intensity within whole single cell were recorded over the next 120 to 200 s, depending on the experimental sample. The fluorescence in the bleached ROI and in the whole cell was quantified at every time point using ImageJ software and the normalized intensity at time *t* was obtained using the equation *F_t = _*(*T_0_*×*B*_t_)+(*T_t_*×*B_0_*), where *F_t_* is the normalized fluorescence in the ROI at time point *t*, *T_0_* and *T_t_* are the fluorescence in the whole cell at time points 0 and *t*, and *B_0_* and *B_t_* are the fluorescence in the bleached ROI at time points 0 and *t*, respectively. Normalized intensity versus time plots were generated using GraphPad Prism 5 software. The mobile (F_M_) and immobile (F_I_) fractions of fluorescent Langerin molecules were estimated with the following equations: *F_M = _*(*I_E_*-*I_0_*)+(*I_I_*-*I_0_*) and *F_I = _*1-*F_M_* , where *I_I_*, *I_E_* and *I_0_* represent the normalized fluorescence at the immediate pre-bleaching time point, the first post-bleaching time point and the plateau, respectively. Means and 95% confidence intervals were calculated with Excel software.

## Results

### Expression of a cytoplasmic FP-tagged Langerin mutant induces the apparition of stacks of Birbeck granule-like structures in M10 cells

**Figure 1:**
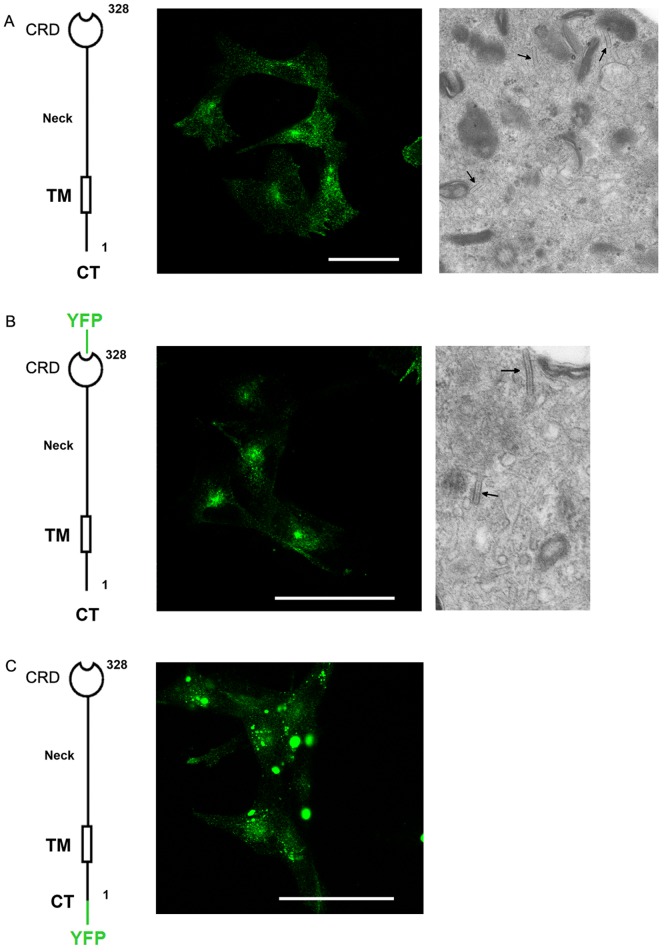
Impact of the position of the YFP tag on the distribution of Langerin. (A) Transfected M10 melanoma cells stably expressing wild type Langerin (M10-22E cells) were fixed, immunolabeled with the anti-CD207 mAb DCGM4 and analyzed by confocal microscopy (left), or processed for electron microscopy (right). (B) Transfected M10 cells stably expressing Lang-YFP were fixed and processed for confocal microscopy analysis of the YFP distribution (left), or for ultrastructural analysis (right). (A and B) Arrows indicate the position of BGs. (C) Transfected M10 cells stably expressing YFP-Lang were fixed and processed for confocal microscopy analysis of the YFP distribution (left). A higher magnification of a region of interest is shown on the right. Scale bars, 50 µm.

Previous observations have demonstrated that the late trafficking steps of C-terminal-tagged Langerin (Lang-YFP) lie under the strict control of a Rab11A membrane organizing platform including myosin Vb and Rab11-FIP2 [19], [20], [25]. This control was also shown to depend on the coordinated physical interactions of the molecular constituents. To further investigate the characteristics of these dynamic interactions, a new YFP-tagged chimeric Langerin protein was generated, having the fluorescent tag in the N-terminal cytosolic position (YFP-Lang) instead of the previous C-terminal luminal/extracellular position (Lang-YFP). The cellular localizations in M10 cells of the two fluorescent fusion molecules were compared to that of wild type Langerin by confocal microscopy. While Lang-YFP and wild type Langerin displayed an identical cellular distribution (**Fig. 1A–B**), YFP-Lang molecules were found to coalesce in enlarged structures (**Fig. 1C**).

**Figure 2:**
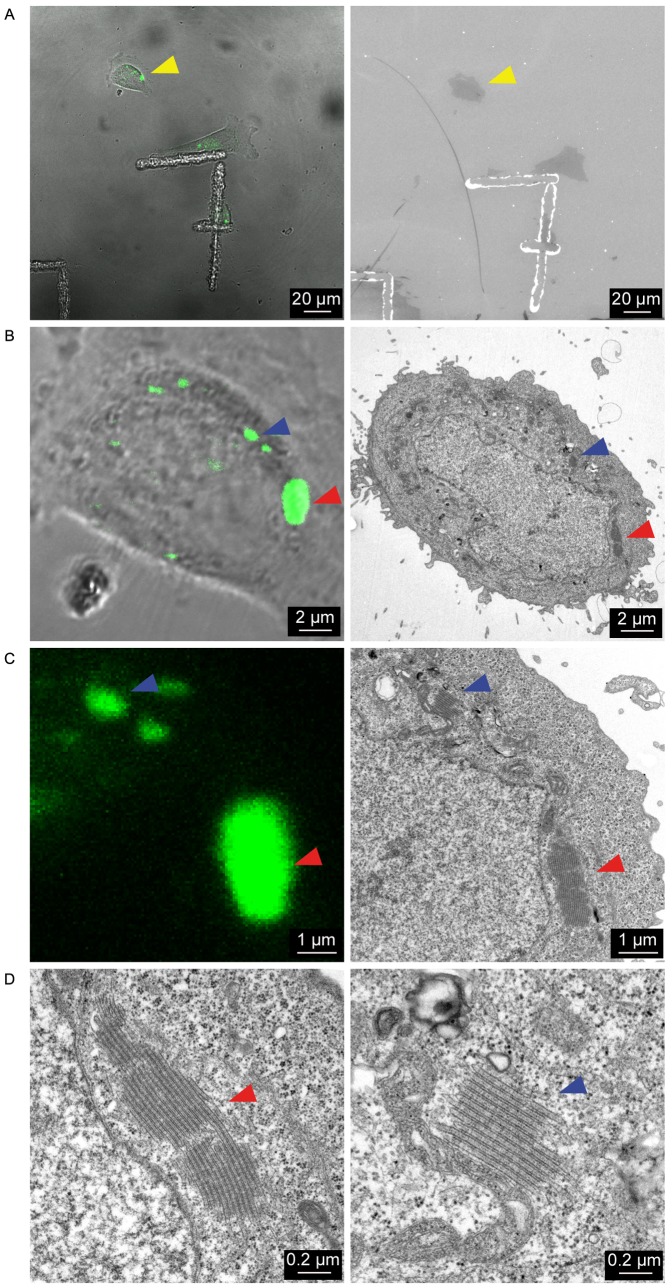
Correlative light-electron microscopy identifies the YFP^+^ puncta as stacks of BG-like membranes. M10-YFP-Lang cells were grown on pre-patterned Aclar® culture supports and the ultrastructure of the YFP^+^ puncta was determined by CLEM. (A) A cell of interest was located by bright field microscopy (left panel) and readily retrieved under the electron microscope (right panel) with the help of the still apparent “7” mark. (B and C) Higher magnifications of the same cell in bright field fluorescent microscopy (left panels) and electron microscopy (right panels), where the fluorescent puncta (blue and red arrowheads) appear as stacks of BG-like membranes (better seen in (D) at a still higher magnification).

To precisely identify the nature of these structures, we employed a CLEM strategy. Using a recently described sample preparation method [23], cells of interest were precisely located on the support by fluorescence and transmission light microscopy (**Fig. 2A**), and then processed for electron microscopy. In this way, we could accurately identify these accumulations of Langerin fluorescence as highly organized stacks of membranes having a pentalaminar structure reminiscent of BGs (**Fig. 2B–D**). Notably, the spaces between the pentalaminar sheets within the stacks were narrow, with a constant width of 8 to 9 nm. Similar structures were observed in M10 cells transiently transfected to express a cytoplasmic GFP-tagged Langerin [26] (**Fig. S1**). Altogether, these results showed that the expression of a cytosolic, but not a luminal, FP-tagged Langerin protein induces the formation of thick stacks of BG-like membranes.

### Ultrastructural studies identify the YFP-Lang-induced BG-like structures as a particular type of OSER

**Figure 3:**
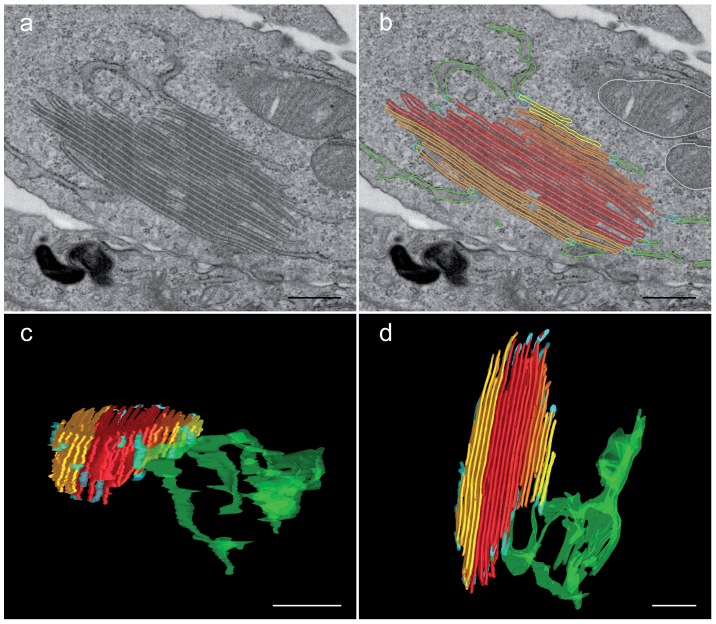
Continuity of the BG-like structures with the rough ER. Transfected M10 cells stably expressing YFP-Lang were processed for electron microscopy. M10 cells stably expressing YFP-Lang were processed for FIB/SEM. The surface of the block was ion-milled and serial images were acquired. A 3D reconstruction was then obtained from the image stack after manual segmentation. (A) A stack of BG-like membranes and sacs of the rough ER in the same plane with (B) manual segmentation of the different objects of interest. The BG-like structures appear in orange, yellow and red and the ER in green. (C and D) A 3D reconstruction demonstrating that continuity (blue segments) exists between the BG-like membranes and the ER (two different angles of view, see also **Video S1**). Scale bars: 500 nm.

Careful examination of ultrathin sections by electron microscopy with tilting revealed that the BG-like membranes were in continuity with the rough ER, although ribosome-free (**Fig. 3A**). These observations were confirmed using FIB/SEM studies followed by 3D reconstructions. Once again, clear connections between the rough ER and the ribosome-free BG-like stacks were observed (**Fig. 3B** **(blue segments), Video S1**). Immunofluorescence staining followed by confocal microscopy imaging showed that these ER-associated structures contained BiP/GRP78 and calnexin (**Fig. S2A**). Nevertheless, electron microscopy analysis of immunogold-labelled cryosections of M10 cells expressing YFP-Lang showed that KDEL-tagged ER-localized endogenous proteins and protein disulfide isomerase were located in ER sacs but not in BG-like membrane stacks, demonstrating that a number of ER resident molecules were excluded from these structures. In contrast, anti-GFP, and -Langerin Abs stained these latter structures (**Fig. S2B**).

To ascertain the retention of YFP-Lang molecules in the ER, solubilized membrane protein extracts were treated with the endoglycosidase PNGase F or endoglycosidase H (EndoH) and analyzed by western blotting with an anti-GFP mAb. The majority of the YFP-Lang molecules were EndoH-sensitive, indicating that they had not egressed from the ER (**Fig. S2C**). Hence these highly organized BG-like stacks of membranes represented Langerin-enriched subcompartments, in continuity with the rough ER but excluding the classical ER-associated chaperone molecules.

### YFP-Lang molecules display a strongly reduced mobility and absence of lateral membrane diffusion within the BG-like OSER

Fluorescent live cell imaging revealed an overall reduced traffic of the YFP-Lang chimera, as compared to the C-terminal-tagged Lang-YFP fusion protein (**Video S2, S3**), consistent with YFP-Lang being retained in the BG-like OSER. To assess the mobility of Langerin molecules within these structures and the putative membrane exchanges with other intracellular pools, FRAP experiments were conducted.

**Figure 4:**
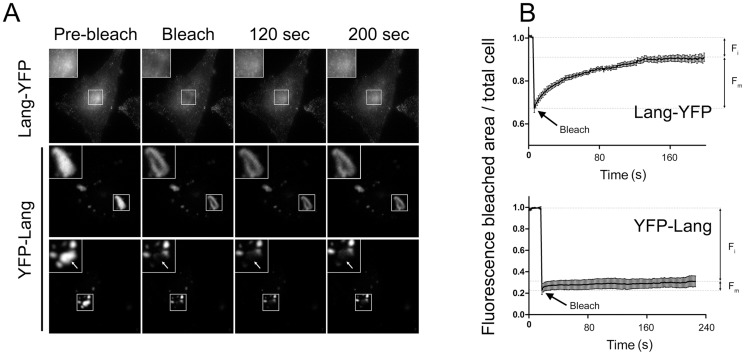
FRAP analysis of the mobilities of Langerin/YFP chimeras. (A) Selected regions of M10 cells stably expressing either Lang-YFP (upper panels) or YFP-Lang (middle and lower panels) were bleached (arrows) and the fluorescence in single z-sections was recorded approximately every 1.6 s. (B) The fluorescence in the bleached area was quantified and plotted against time after correction for the change in total fluorescence. At least 10 cells were analyzed for each plot; error bars indicate the standard error of the mean. F_i_ and F_m_ designate the immobile and mobile fractions of the Langerin/YFP chimeras, respectively.

Complete structures or a part of them, accumulating YFP-Lang (**Fig. 4A**, middle and lower panels), were photobleached and the fluorescence recovery was recorded. There was virtually no fluorescence recovery of YFP-Lang in both cases, indicating not only a relative absence of lateral diffusion of YFP-Lang molecules within the enlarged structures, but also a lack of active molecular exchanges with any other pools within the cells, including the rough ER membranes. In similar experiments in M10-Lang-YFP cells (**Fig. 4A**, upper panel), the bleached areas corresponded to the pericentriolar regions, where fluorescent Langerin appeared to rapidly re-accumulate. Thus, quantification of the mobile fraction (F_m_) of chimeric molecules showed that less than 12±8% (95% confidence interval) of the YFP-Lang signal could be recovered within 4 min after photobleaching, whereas 69±12% (95% confidence interval) of Lang-YFP molecules rapidly repopulated the pericentriolar region, stabilizing at a plateau level within 2 min (**Fig. 4B**). Very likely, this apparent immobilization probably reflects tight interactions between YFP-Lang fusion proteins. In order to clarify the respective roles of the luminal lectin domains, located within the BG-like OSER, and the cytoplasmic domains, present in the spaces separating the stacks of adjacent membranes, additional molecular information was required.

### Homotypic interactions between cytoplasmic YFP tags are strongly involved in the biogenesis of BG-like OSER

As already mentioned, the CRD of the Langerin luminal domain is critical for the zipping process inducing the appearance of BGs [18]. However, this does not by itself explain the biophysical particularities observed here for YFP-Lang-induced stacking. Since GFP has an intrinsic ability to dimerize, forming an anti-parallel orientation [27], we hypothesized that this dimerization was responsible for crosslinking of the cytoplasmic domains of YFP-Lang molecules, thus contributing to the stacking of the BG-like pentalaminar structures and to the ER retention of YFP-Lang and its frozen dynamics in our model. To explore this hypothesis, we introduced an A206K substitution into the YFP sequence [4] and expressed a cytoplasmic monomeric (m)YFP-tagged Langerin chimera (mYFP-Lang) in M10 cells.

**Figure 5:**
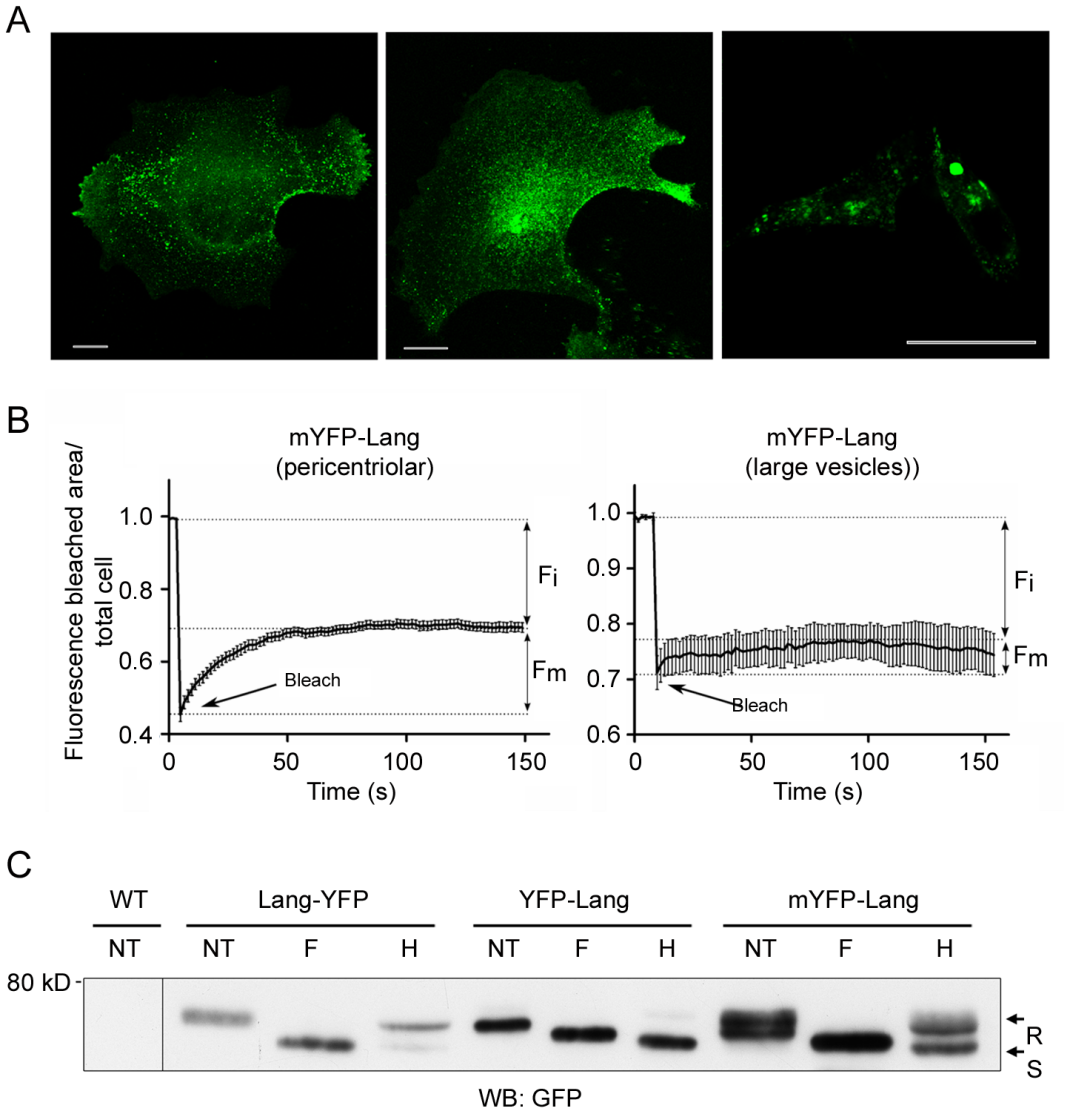
A monomerizing substitution in YFP impairs the formation of BG-like OSER and restores BG dynamics. (A) M10 cells stably expressing mYFP-Lang were fixed and the YFP distribution was examined by confocal microscopy. Overlays of 5–10 z-sections from different representative cells are depicted. Left and middle panels: images representative of the majority of cells, with a pericentriolar concentration of fluorescence and small vesicles dispersed across the cytoplasm. Right panel: YFP^+^ puncta present only in a minority of cells. Scale bars: 25 µm. (B) FRAP experiments were carried out as in **Fig. 5B**. Left panel: to study the fluorescence recovery in the pericentriolar region, 16 cells were analyzed for each plot. Right panel: to study the fluorescence recovery in enlarged vesicles, 8 cells were analyzed for each plot. Error bars represent the standard error of the mean. F_i_ and F_m_ indicate the immobile and mobile fractions of mYFP-Lang molecules, respectively. (C) Soluble membrane extracts (30 µg) of M10 cells stably expressing transgenic Lang-YFP, YFP-Lang or mYFP-Lang were digested or not (NT) with PNGaseF (F) or endoglycosidase Hf (H) and separated by 7.5% SDS-PAGE. Fusion proteins were revealed by western blotting with an anti-GFP antibody. R and S indicate endoglycosidase Hf-resistant and sensitive species, respectively. Untransfected cells (WT) were used as a control.

In most of the stably transfected cells, the distribution of the YFP fluorescence was very similar to that observed in M10-Lang-YFP cells (**Figs. 5A** **and S3b and b2**), i.e., a pericentriolar concentration of the fluorescence with small vesicles dispersed across the cell. Although a few cells with larger vesicles were also identified (less than 10%) (**Figs. S3a and a2**), all our attempts to subclone cells with this peculiar phenotype remained unsuccessful. Ultrastructural examination using CLEM revealed the presence of “classical” Birbeck granules, either in the pericentriolar region or beneath the plasma membrane (**Fig. S3b2**). Consistently, BG-like OSER was also observed in the rare cells still displaying fluorescent puncta (**Fig. S3a2**). Fluorescent live cell imaging revealed restoration of the traffic of mYFP-Lang molecules (**Video S4**, **Fig. S4**). FRAP experiments confirmed that the mobility of these YFP-Lang fusion proteins within the pericentriolar region was partially restored, with a mobile fraction (F_m_) of 43.7±5.1% (confidence interval, 95%) (**Fig. 5B** **, left panel**). In contrast, this mobility was still strongly impaired in the more rarely observed enlarged vesicles, with an F_m_ of 23±8.6% (confidence interval, 95%) (**Fig. 5B** **, right panel**).

Finally, the ability of mYFP-Lang molecules to egress from the ER was compared to that of Lang-YFP and YFP-Lang molecules by means of biochemical experiments. Endoglycosidase digestion revealed that the monomerizing mutation of YFP partially restored the ER egression capacity of the cytoplasmic-tagged Langerin protein (**Fig. 5C**).

Altogether, these results showed that YFP-YFP dimerization was directly involved in the stacking mechanism leading to the formation of BG-like OSER.

### An intact calcium binding site on Langerin molecules is also required for the formation of OSERs

**Figure 6:**
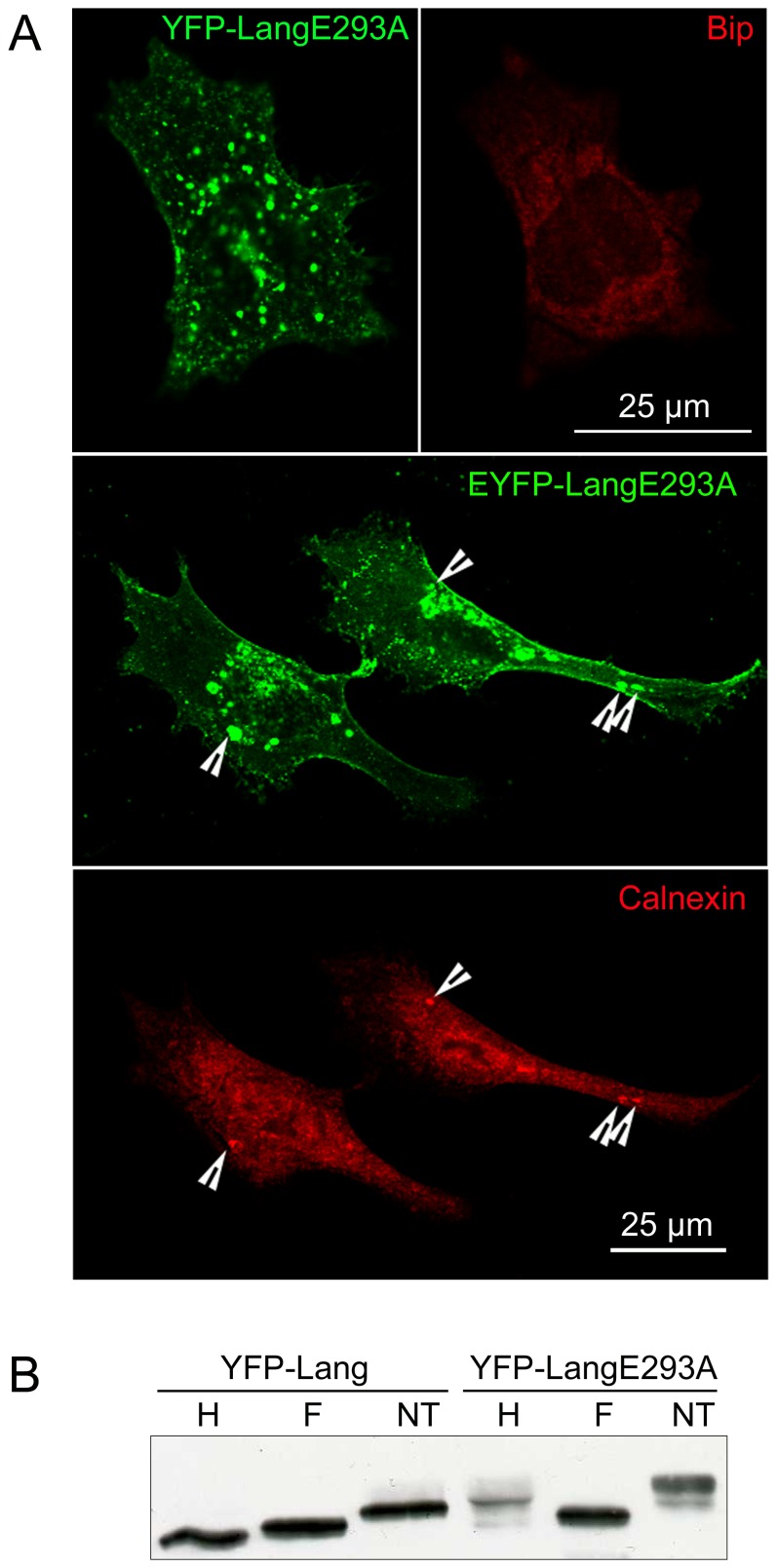
Mutation of the calcium binding domain does not block OSER formation, but restores the dynamic transport of YFP-Langerin mutant. (A) M10 cells stably expressing YFP-LangE293A were fixed, stained with anti-calnexin and anti-Bip antibodies and analyzed by confocal microscopy. (B) EnodH resistance of YFP-LangE293A molecules was analyzed as described in Fig. 5C.

In order to better understand the role of Langerin CRD in the formation of immobile BG-like OSERs, a site directed mutagenesis experiment was performed. The CRD of Langerin is structurally homologous to site CRD-4 in the macrophage mannose receptor (MMR) [28]. This site has been well characterized, four key amino acids allow the binding of calcium ions and E733A mutation affects the binding of calcium and of mannose on MMR [29]. The amino acids that coordinate calcium in MMR CRD-4 are conserved in Langerin; in particular, E733 on MMR corresponds to E293 on Langerin. Consequently, an EYFP-Langerin E293A mutant was expressed in M10 cells. Immunofluorescence confocal microscopy showed that this protein accumulated under (near or at) the plasma membrane and, in addition, in rather large structures that could be as large as 2–4 µm^2^ (Fig. 6A). Interestingly, YFP-LangE293A positive compartments were not stained by anti-calnexin Ab, while occasional and partial colocalization with Bip was noticed (**Fig. 6A**). Most of YFP-LangerinE293A molecules were EndoH-resistant and, appeared more heavily glycosylated than the ER-retained YFP-Lang molecules (**Fig. 6B**) and thus, had left the ER. Electron microscopy analysis of sections of Epon™ embedded cells (Fig. S5) and, of immunolabeled cryosections (Fig. S6) revealed the presence of large double membrane structures. They occasionally appeared to extend over several µm, either as intracellular membranes (Fig. S6b), or as contacts between two adjacent cells (Fig. S6c). Most often, the double membranes looked like sections of vesicles or invaginations, the diameter of which could exceed 500 nm (Fig. S5), even 1 µm (Fig. S6e). Of note, the enclosed material appeared to be cytosolic, containing ribosomes (Fig. S5). The central striation characteristic to BG could be occasionally observed (**Fig. S5f**) but most often, was faint (**Fig. S5a**) or could not be clearly ascertained (**Fig. S5b, c, d, e**). Due to these properties of the central striation and, to facilitate the report and the discussion, we propose to name these structures pseudo-BGs. A first major difference between pseudo-BGs and usual BGs is the large size of the pseudo-BGs. A second one is the apparent “concentric” arrangement of the membranes, as seen in Fig. S5a and S6f, which after folding could generate a more complex pattern (Fig. S6d). Immunogold labeling confirmed the presence of membrane anchored YFP proteins in pseudo-BGs (**Fig. S6**). Between them, pseudo-BGs could form close contacts that were immunolabeled by anti-YFP Abs (Fig. S6d, f), indicating that homodimerization of YFP molecules still occurred and could mediate membrane apposition. Although the DCGM4 stained LangE293A using immunofluorescence techniques (data not shown), in immune-electron microscopy it was very poorly efficient; occasionally, pseudo-BGs were stained by one or two gold particles (data not shown).

Altogether, these observations show that the mutation of the CRD allowed the formation of large double membrane structures, with optional central striations, which did not belong to the ER but could occasionally be in contact with another similar double membrane structure.

## Discussion

This paper illustrates the potency of correlative microscopy using FIB/SEM analysis, which allowed to study large cellular structures extending within a volume of several µm^3^ and, cannot be obtained with the same accuracy and easiness using TEM combined with tomography. The artificial induction of stacks of BGs by an FP-tagged Langerin mutant described here is a good illustration of the view that BGs are generated “wherever Langerin accumulates”. This can be also seen (i) in the early endosomal recycling compartments in the steady state [16], [22], (ii) in the tubular networks emanating from the endosomal recycling compartments when Langerin recycling is inhibited [16], (iii) on the plasma membrane of cells treated with inhibitors of endocytosis [30], or (iv) in the multivesicular compartments of cells treated with inhibitors of lysosomal degradation [25].

Our observations are compatible with those of Verdijk et al, who expressed an N-terminal cytoplasmic GFP-tagged Langerin mutant in human fibroblastic cells [26]. Although fluorescent puncta (corresponding to BG-like OSER) were not described by these authors, stacks of BG-like membranes were nevertheless present, as attested by electron microscopy (Fig. 2 of Verdijk's publication). We confirmed that transient expression of this fusion protein in the M10 cell line also resulted in the appearance of BG-like OSER (**Fig. S1**). In addition, careful re-examination of Fig. 2B of Verdijk's publication strongly suggests the existence of continuity between a BG and a flattened cisterna of the rough ER, as we observed in the case of YFP-Lang-induced BG-like OSER.

The CRD of Langerin is crucial for the membrane zipping process which occurs during BG biogenesis [18] and hence the expression of YFP-Lang was expected to induce the formation of BG-like pentalaminar structures. Surprisingly, while expression of Lang-YFP induced the formation of *bona fide* BGs (11, 17), confocal microscopy analyses revealed that the expression of YFP-Lang resulted in the formation of large Bip^+^ or calnexin^+^ YFP^+^ puncta, indicating that the chimera was retained in the ER. In agreement with this conclusion, YFP-Lang molecules remained EndoH sensitive. Nevertheless, the ER resident endogenous proteins, protein disulfide isomerase and KDEL-tagged proteins were excluded from these structures. Correlative electron microscopy revealed they were stacks of BG-like membranes, which did not belong to the endosomal network, but rather were in continuity with the rough ER. These stacks were characterized by narrow cytoplasmic spaces of an almost constant width of 8–9 nm, compatible with, if not characteristic to, the existence of direct interaction bridges between proteins in the apposed membranes. Altogether, we concluded that the expression on YFP-Lang, induces the formation of a particular form of OSER. One distinctive property of YFP-Lang-induced OSER was their strong immobility, in contrast to GFP-tagged cytochrome b5, an ER resident protein, which appeared to diffuse rapidly in and out of the OSER it induced [5]. Of note, these latter OSER were stained by anti-protein disulfide isomerase, contrary to what we observed in our system. A remarkable feature of the YFP-Lang-induced OSER was topologic, as they appeared as stacks of membrane sheets that clearly did not display the symmetry and the periodicity of cubic membranes [12].

While the CRD of Langerin is responsible for the biogenesis of BGs, the cytosolic YFP tag mediated the stacking of the BGs and their “transformation” into OSER, similarly to what has been already described [5]. This latter property has been attributed to the anti-parallel homodimerization of these cytoplasmic fluorescent YFP/GFP tags, which can be avoided by using a monomeric A206K substituted protein [5]. The use of this monomeric YFP mutant indeed almost completely suppressed the formation of YFP-Lang-induced OSER, strongly supporting the view that they were largely dependent on a membrane tethering mediated by YFP homodimerization, although it cannot be excluded that interactions between YFP and Langerin cytoplasmic tail also contribute to OSER formation. The importance of YFP tag in the generation of BG stacks within the smooth ER is confirmed by the work of Thepaut et al who showed that a 28 amino acid deletion of Langerin cytoplasmic domain allowed the formation BG-like structures within the rough ER, without displaying the striking stacking reported here in the smooth ER. Induction of OSER by overexpressed ER-resident proteins, tagged or not, is occasionally observed but well documented [5]–[8]. A specifi role of GFP tag has been formally demonstrated in a few examples of tagged proteins, Sec61β, Sec61γ and a C-terminal peptide of P450 cytochrome [5]. OSER formation was found to depend on the ability of the (fused) protein to oligomerize, which is also the case of Langerin, since it is trimeric [28] and can form homotypic interactions between the luminal domains facing one to the other on apposed membranes.

To better evaluate the role of these homotypic interactions, we tested the E293A mutation, which is analogous to E733A mutation in MMR. In the mannose receptor, E733A mutation affects the binding of both calcium and mannose [29]. Electron microscopy experiments revealed that YFP-LangE293A can still induce the formation of intracellular double membrane structures. Such structures could be noticed between membranes bridging two adjacent cells (Fig. S6c), as observed when wild type Langerin is expressed (unpublished observations). The large size of the induced intracellular double membrane structures and the optional presence of central striation prompt us to consider these structures as pseudo-BGs. The effect of the mutation on the central striation suggests that the affinity of calcium to LangE293A and the ability of the mutated lectin to form homodimers are dumped. These properties need to be investigated in details.

The inefficiency of LangE293A to form homodimers via their CRD is indirectly supported by the biochemical and cellular properties specific to YFP-LangE293A molecules. Firstly, contrary to wild type YFP-Lang, at the steady state most of YFP-LangE293A molecules were EndoH-resistant, which means that they left the ER. Secondly, in agreement with this observation, most of YFP-LangE293A molecules accumulate in membrane compartments that were not labeled with anti-Bip and anti-calnexin Abs. Thirdly, electron microscope analysis revealed that these structures accumulated in large double membrane sheets. Notably, sections showed that the membrane structures induced delineated ribosome containing spaces, suggesting that they were sections of invaginated large intracellular membranes. Local membrane coupling could be observed (**Fig. S6d, 6f**), suggesting that YFP was able to mediate the coupling between the cytosolic faces of the double membranes. Hence, YFP-LangE293A molecules accumulated in intracellular membranes that do not belong to the ER network and, did not form OSER-like structures.

**Figure 7:**
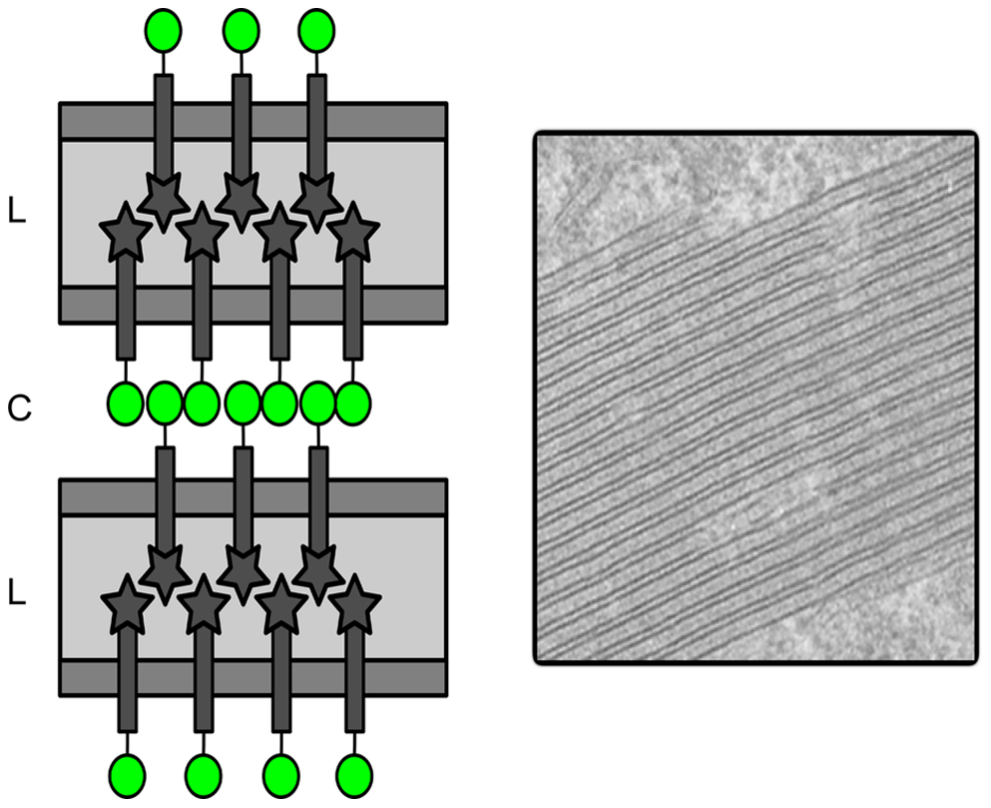
Schematic view of the proposed mechanism of formation of BG-like OSER. The ER lumen (L) is depicted in pale gray and the cytoplasm (C) in white. Homotypic interactions between the CRD domains of Langerin (C-terminal, intra-luminal position, dark gray stars) are responsible for the luminal zipping of Langerin-enriched ER membranes, while homotypic interactions between YFP molecules (N-terminal, cytoplasmic position, green circles) are involved in the stacking of BG-like membranes. This “two-lock mechanism” could plausibly explain the high rigidity and immobility of these structures.

The BG-like OSER displays unique properties, which can probably be explained by the synergy of two kinds of homotypic interaction: (i) CRD-CRD interactions in the lumen of the OSER, responsible for the BG-like pentalaminar appearance, and (ii) YFP-YFP interactions in the cytoplasmic spaces separating the apposed membranes, responsible for their stacking. Of note, the trimeric structure of Langerin [28] may also contribute to the immobility of the EYFP-Lang molecules in the OSERs. A “double-lock” mechanism would therefore be at the origin of this particular type of OSER (**Fig. 7**). This model is reminiscent of the OSER structures induced by J13Lp protein of African swine fever virus; its cytoplasmic tail mediated the formation of OSER, while inter-disulfide bonds between the luminal domains of the protein induced the collapse of the ER cisternae. Together, the interactions induced the formation of stacked and collapsed OSER. However, when J13Lp protein was mutated in order to block the formation of intermolecular di-sulfide bonds, ER cisternae did not collapse anymore, although OSER still formed [31]. In contrast, the E293A mutation resulted in the absence of OSER formation but, in the generation of complex membrane organizations unrelated to the ER. The inability of the YFP cytoplasmic tag to induce OSER might be explained by the presence of dominant cellular transport mechanisms particular to Langerin and further investigations will be necessary to clarify this feature. Overall, this work based on a simple model of a unique molecule, underlies the importance of combined molecular interactions in the modulation of the topology of intracellular membranes and hence, biogenesis of membrane compartments.

## Supporting Information

Figure S1 **Stacks of BG-like membranes are also induced by a GFP-Lang fusion protein.** A GFP-Lang fusion protein was transiently expressed in M10 cells. (A) A GFP^+^ cell (yellow arrow) was located by fluorescence microscopy (left panel), bright field microscopy (middle panel) and electron microscopy (right panel). (B) GFP^+^ puncta (red, blue and orange arrowheads) located by bright field microscopy (left panel, higher magnification) were retrieved in ultrathin sections (corresponding to different Z) by electron microscopy (middle and right panels). (C and D) Higher magnifications of the fluorescence microscopy (left panels) and electron microscopy (middle and right panels) acquisitions identified these puncta as stacks of BG-like membranes. Continuity with the rough ER is suggested (right panels, arrowheads).
(TIF)

Figure S2 **Characterization of the BG-like structures.** (A) The presence of the ER chaperones BiP and calnexin (CNX) in YFP^+^ large puncta was studied in colocalization experiments. M10-YFP-Lang cells were fixed, permeabilized and stained with rabbit anti-BiP or anti-calnexin Abs or an isotype control (revealed with Cy5-conjugated donkey anti-rabbit Abs, red). Colocalization with YFP (green) is depicted in yellow. Arrows indicate exclusion of the BiP immunostaining from large YFP^+^ structures. Scale bars: 25 µm. (B) M10 cells expressing YFP-Lang were processed for cryoelectron microscopy and immunolabeled with antibodies specific for GFP, Langerin, KDEL peptide (KDEL) or protein disulfide isomerase (PDI). Pictures of anti-GFP and anti-Langerin staining of BG-like membrane stacks (upper panels) and anti-KDEL and anti-PDI labeling of BG-like membrane stacks (right) and ER structures (left) are shown.(C) Solubilized membrane protein extracts (10 µg) of M10-YFP-Lang or untransfected (WT) cells were digested or not (NT) with PNGase F (F) or endoglycosidase Hf (EndoH, H) and separated by 7.5% SDS-PAGE. YFP-tagged molecules were revealed by western blotting using an HRP-conjugated anti-GFP Ab. R and S indicate EndoH-resistant and sensitive species, respectively.
(TIF)

Figure S3 **CLEM analysis of cells expressing mYFP-Lang.** M10 cells expressing mYFP-Lang were processed for CLEM as in **Fig. 2**. On the same Aclar® culture support, two cells with different phenotypes were observed: the first (a, a2, yellow arrowhead) displayed small puncta which were identified ultrastructurally as BG-like OSER; the second (b, b2, blue arrowhead) displayed classical, pericentriolar rod-shaped BGs.
(TIF)

Figure S4 **Restoration of the mobility of YFP-Lang with the A206K monomerizing mutant of YFP.** Maximum intensity projections, generated from t-stacks of images acquired during FRAP experiments, are depicted for M10-Lang-YFP (left panel), M10-YFP-Lang (middle panel) and M10-mYFP-Lang (right panel) cells. The mobility of the Langerin/YFP chimeras can be roughly estimated from the presence of elongated, linear structures corresponding to small vesicles in motion, particularly visible in the immediate proximity of the plasma membrane or in the pericentriolar region (arrows). These elongated structures are nearly absent in M10-YFP-Lang cells, but similarly present in M10-Lang-YFP and M10-mYFP-Lang cells.
(TIF)

Figure S5 **M10 transfected cells expressing YFP-LangE293A were fixed included in Epon.** Sometimes, the central striation characteristical to classical BGs were noticed (white arrows).
(TIF)

Figure S6 **M10 transfected cells expressing YFP-LangE293A were fixed with 0.2% gluteraldehyde 2% paraformaldehyde, frozen in liquid N2, cryosections were labeled with rabbit polyclonal anti-GFP Abs, revealed with protein A conjugated 10 nM gold particles (PAG, Utrecht) and analyzed on CM120 electronic microscope (FEI).**
(TIF)

Video S1 **A 3D reconstruction of a stack of BG-like membranes viewed with FIB/SEM, demonstrating continuity with the rough ER (same cell as in** **Fig. 4** **).**
(AVI)

Videos S2 **Images acquired during FRAP experiments on transfected M10 cells expressing Lang-YFP cells.** Multiple fluorescent vesicles can be seen in motion.
(AVI)

Video S3 **Images acquired during FRAP experiments on transfected M10 cells expressing YFP-Lang cells.** The fluorescent structures are nearly motionless.
(AVI)

Video S4 **Images acquired during FRAP experiments on transfected M10 cells expressing mYFP-Lang.** The introduction of the A206K mutation restores the mobility of the fluorescent vesicles.
(AVI)

## References

[1] Shimomura O, Johnson FH, Saiga Y (1962) Extraction, purification and properties of aequorin, a bioluminescent protein from the luminous hydromedusan, Aequorea. J Cell Comp Physiol 59: 223–239. 13911999 10.1002/jcp.1030590302

[2] Prasher DC, Eckenrode VK, Ward WW, Prendergast FG, Cormier MJ (1992) Primary structure of the Aequorea victoria green-fluorescent protein. Gene 111: 229–233. 1347277 10.1016/0378-1119(92)90691-h

[3] Snapp EL (2009) Fluorescent proteins: a cell biologist's user guide. Trends Cell Biol 19: 649–655. 19819147 10.1016/j.tcb.2009.08.002 PMC2784028

[4] Zacharias DA, Violin JD, Newton AC, Tsien RY (2002) Partitioning of lipid-modified monomeric GFPs into membrane microdomains of live cells. Science 296: 913–916. 11988576 10.1126/science.1068539

[5] Snapp EL, Hegde RS, Francolini M, Lombardo F, Colombo S, et al (2003) Formation of stacked ER cisternae by low affinity protein interactions. J Cell Biol 163: 257–269. 14581454 10.1083/jcb.200306020 PMC2173526

[6] Parrish ML, Sengstag C, Rine JD, Wright RL (1995) Identification of the sequences in HMG-CoA reductase required for karmellae assembly. Mol Biol Cell 6: 1535–1547. 8589454 10.1091/mbc.6.11.1535 PMC301309

[7] Volkova EG, Abramchuk SS, Sheval EV (2012) The overexpression of nuclear envelope protein Lap2beta induces endoplasmic reticulum reorganisation via membrane stacking. Biol Open 1: 802–805. 23213473 10.1242/bio.20121537 PMC3507222

[8] Korkhov VM, Zuber B (2009) Direct observation of molecular arrays in the organized smooth endoplasmic reticulum. BMC Cell Biol 10: 59. 19703297 10.1186/1471-2121-10-59 PMC2737311

[9] Fasana E, Fossati M, Ruggiano A, Brambillasca S, Hoogenraad CC, et al (2010) A VAPB mutant linked to amyotrophic lateral sclerosis generates a novel form of organized smooth endoplasmic reticulum. FASEB J 24: 1419–1430. 20008544 10.1096/fj.09-147850

[10] Lingwood D, Schuck S, Ferguson C, Gerl MJ, Simons K (2009) Generation of cubic membranes by controlled homotypic interaction of membrane proteins in the endoplasmic reticulum. J Biol Chem 284: 12041–12048. 19258319 10.1074/jbc.M900220200 PMC2673273

[11] Almsherqi ZA, Kohlwein SD, Deng Y (2006) Cubic membranes: a legend beyond the Flatland* of cell membrane organization. J Cell Biol 173: 839–844. 16785319 10.1083/jcb.200603055 PMC2063909

[12] Almsherqi ZA, Landh T, Kohlwein SD, Deng Y (2009) Chapter 6: cubic membranes the missing dimension of cell membrane organization. Int Rev Cell Mol Biol 274: 275–342. 19349040 10.1016/S1937-6448(08)02006-6 PMC7105030

[13] Valladeau J, Ravel O, Dezutter-Dambuyant C, Moore K, Kleijmeer M, et al (2000) Langerin, a novel C-type lectin specific to Langerhans cells, is an endocytic receptor that induces the formation of Birbeck granules. Immunity 12: 71–81. 10661407 10.1016/s1074-7613(00)80160-0

[14] Hunger RE, Sieling PA, Ochoa MT, Sugaya M, Burdick AE, et al (2004) Langerhans cells utilize CD1a and langerin to efficiently present nonpeptide antigens to T cells. J Clin Invest 113: 701–708. 14991068 10.1172/JCI19655 PMC351318

[15] van der Vlist M, de Witte L, de Vries RD, Litjens M, de Jong MA, et al (2011) Human Langerhans cells capture measles virus through Langerin and present viral antigens to CD4(+) T cells but are incapable of cross-presentation. Eur J Immunol 41: 2619–2631. 21739428 10.1002/eji.201041305

[16] Mc Dermott R, Ziylan U, Spehner D, Bausinger H, Lipsker D, et al (2002) Birbeck granules are subdomains of endosomal recycling compartment in human epidermal Langerhans cells, which form where Langerin accumulates. Mol Biol Cell 13: 317–335. 11809842 10.1091/mbc.01-06-0300 PMC65091

[17] Birbeck MS, Breathnach AS, Averall JD (1961) An electron microscope study of basal melanocytes and high-level clear cells (Langerhans cells) in vitiligo. J Invest Dermatol 37: 51–63.

[18] Thepaut M, Valladeau J, Nurisso A, Kahn R, Arnou B, et al (2009) Structural studies of langerin and Birbeck granule: a macromolecular organization model. Biochemistry 48: 2684–2698. 19175323 10.1021/bi802151w

[19] Boulanger J, Gidon A, Kervran C, Salamero J (2010) A patch-based method for repetitive and transient event detection in fluorescence imaging. PLoS One 5: e13190. 20976222 10.1371/journal.pone.0013190 PMC2955530

[20] Gidon A, Bardin S, Cinquin B, Boulanger J, Waharte F, et al (2012) A Rab11A/Myosin Vb/Rab11-FIP2 Complex Frames Two Late Recycling Steps of Langerin from the ERC to the Plasma Membrane. Traffic 13: 815–833. 22420646 10.1111/j.1600-0854.2012.01354.x

[21] Mackensen A, Ferradini L, Carcelain G, Triebel F, Faure F, et al (1993) Evidence for in situ amplification of cytotoxic T-lymphocytes with antitumor activity in a human regressive melanoma. Cancer Res 53: 3569–3573. 8339262

[22] McDermott R, Bausinger H, Fricker D, Spehner D, Proamer F, et al (2004) Reproduction of Langerin/CD207 traffic and Birbeck granule formation in a human cell line model. The Journal of investigative dermatology 123: 72–77. 15191545 10.1111/j.0022-202X.2004.22728.x

[23] Spiegelhalter C, Tosch V, Hentsch D, Koch M, Kessler P, et al (2010) From dynamic live cell imaging to 3D ultrastructure: novel integrated methods for high pressure freezing and correlative light-electron microscopy. PLoS One 5: e9014. 20140253 10.1371/journal.pone.0009014 PMC2815783

[24] Kremer JR, Mastronarde DN, McIntosh JR (1996) Computer visualization of three-dimensional image data using IMOD. J Struct Biol 116: 71–76. 8742726 10.1006/jsbi.1996.0013

[25] Uzan-Gafsou S, Bausinger H, Proamer F, Monier S, Lipsker D, et al (2007) Rab11A controls the biogenesis of Birbeck granules by regulating Langerin recycling and stability. Molecular biology of the cell 18: 3169–3179. 17538027 10.1091/mbc.E06-09-0779 PMC1949377

[26] Verdijk P, Dijkman R, Plasmeijer EI, Mulder AA, Zoutman WH, et al (2005) A lack of Birbeck granules in Langerhans cells is associated with a naturally occurring point mutation in the human Langerin gene. J Invest Dermatol 124: 714–717. 15816828 10.1111/j.0022-202X.2005.23645.x

[27] Yang F, Moss LG, Phillips GN Jr (1996) The molecular structure of green fluorescent protein. Nat Biotechnol 14: 1246–1251. 9631087 10.1038/nbt1096-1246

[28] Feinberg H, Powlesland AS, Taylor ME, Weis WI (2010) Trimeric structure of langerin. J Biol Chem 285: 13285–13293. 20181944 10.1074/jbc.M109.086058 PMC2857130

[29] Mullin NP, Hitchen PG, Taylor ME (1997) Mechanism of Ca2+ and monosaccharide binding to a C-type carbohydrate-recognition domain of the macrophage mannose receptor. J Biol Chem 272: 5668–5681. 9038177 10.1074/jbc.272.9.5668

[30] Hanau D, Fabre M, Schmitt DA, Garaud JC, Pauly G, et al (1988) Appearance of Birbeck granule-like structures in anti-T6 antibody-treated human epidermal Langerhans cells. The Journal of investigative dermatology 90: 298–304. 3258000 10.1111/1523-1747.ep12456083

[31] Windsor M, Hawes P, Monaghan P, Snapp E, Salas ML, et al (2012) Mechanism of collapse of endoplasmic reticulum cisternae during African swine fever virus infection. Traffic 13: 30–42. 21951707 10.1111/j.1600-0854.2011.01293.x PMC3237792

