# Supplementary figures and images for: Birbeck Granule-Like “Organized Smooth Endoplasmic Reticulum” Resulting from the Expression of a Cytoplasmic YFP-Tagged Langerin
Source: PLoS One. 2013 Apr 5;8(4):e60813. doi: 10.1371/journal.pone.0060813 (PMC3618057; doi:10.1371/journal.pone.0060813)

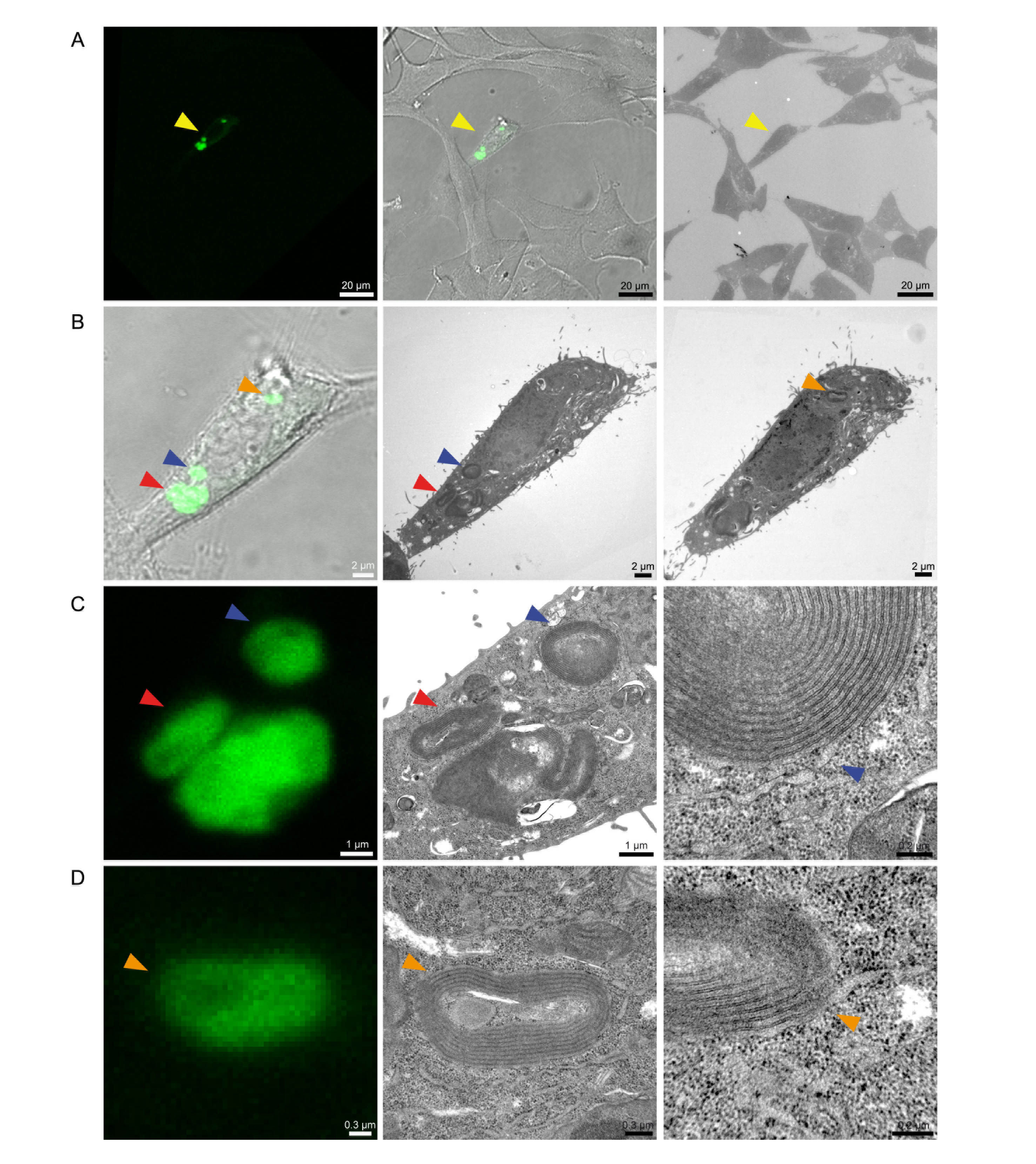

Supplement: Figure S1 — Stacks of BG-like membranes are also induced by a GFP-Lang fusion protein. A GFP-Lang fusion protein was transiently expressed in M10 cells. (A) A GFP+ cell (yellow arrow) was located by fluorescence microscopy (left panel), bright field microscopy (middle panel) and electron microscopy (right panel). (B) GFP+ puncta (red, blue and orange arrowheads) located by bright field microscopy (left panel, higher magnification) were retrieved in ultrathin sections (corresponding to different Z) by electron microscopy (middle and right panels). (C and D) Higher magnifications of the fluorescence microscopy (left panels) and electron microscopy (middle and right panels) acquisitions identified these puncta as stacks of BG-like membranes. Continuity with the rough ER is suggested (right panels, arrowheads). (TIF) [file pone.0060813.s001.tif]

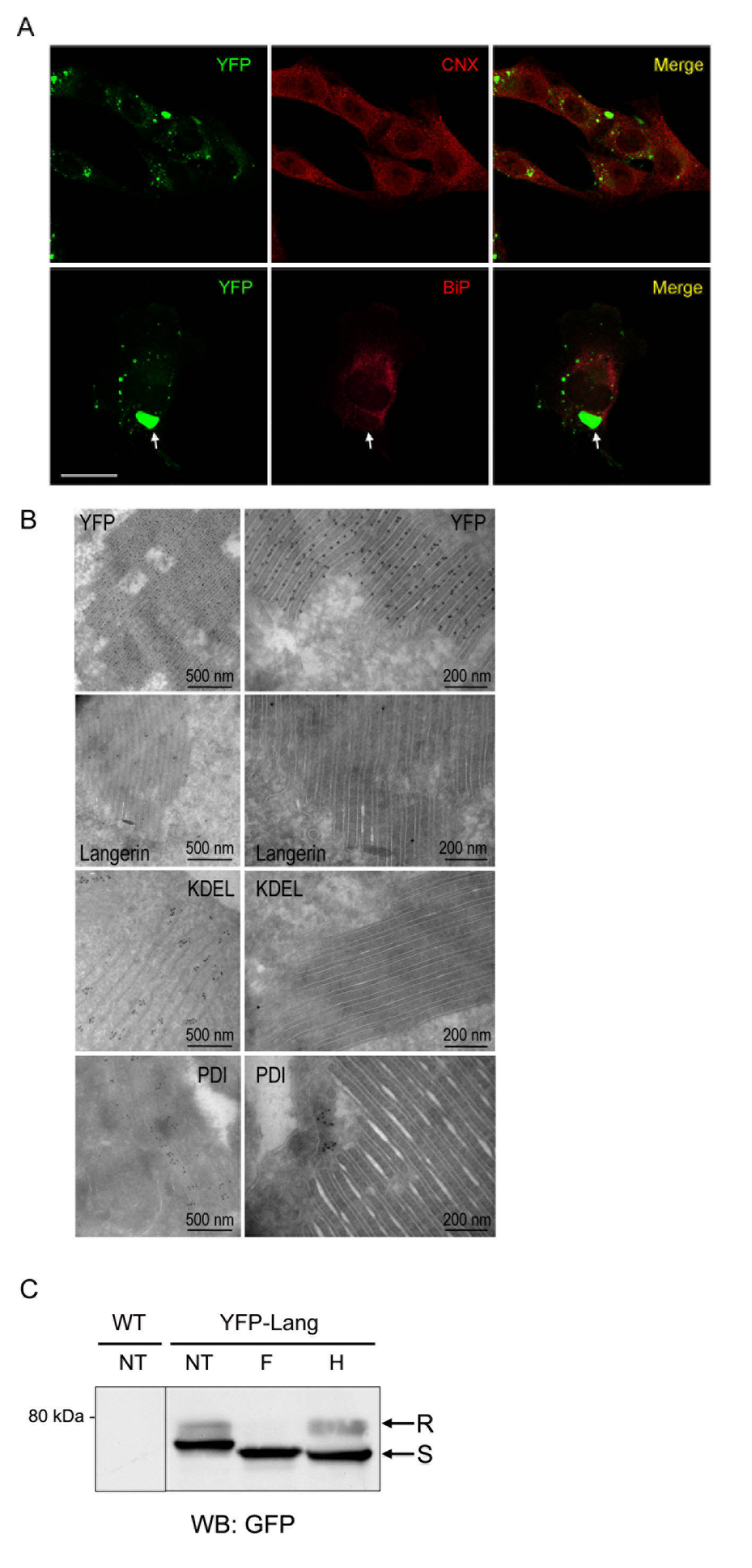

Supplement: Figure S2 — Characterization of the BG-like structures. (A) The presence of the ER chaperones BiP and calnexin (CNX) in YFP+ large puncta was studied in colocalization experiments. M10-YFP-Lang cells were fixed, permeabilized and stained with rabbit anti-BiP or anti-calnexin Abs or an isotype control (revealed with Cy5-conjugated donkey anti-rabbit Abs, red). Colocalization with YFP (green) is depicted in yellow. Arrows indicate exclusion of the BiP immunostaining from large YFP+ structures. Scale bars: 25 µm. (B) M10 cells expressing YFP-Lang were processed for cryoelectron microscopy and immunolabeled with antibodies specific for GFP, Langerin, KDEL peptide (KDEL) or protein disulfide isomerase (PDI). Pictures of anti-GFP and anti-Langerin staining of BG-like membrane stacks (upper panels) and anti-KDEL and anti-PDI labeling of BG-like membrane stacks (right) and ER structures (left) are shown.(C) Solubilized membrane protein extracts (10 µg) of M10-YFP-Lang or untransfected (WT) cells were digested or not (NT) with PNGase F (F) or endoglycosidase Hf (EndoH, H) and separated by 7.5% SDS-PAGE. YFP-tagged molecules were revealed by western blotting using an HRP-conjugated anti-GFP Ab. R and S indicate EndoH-resistant and sensitive species, respectively. (TIF) [file pone.0060813.s002.tif]

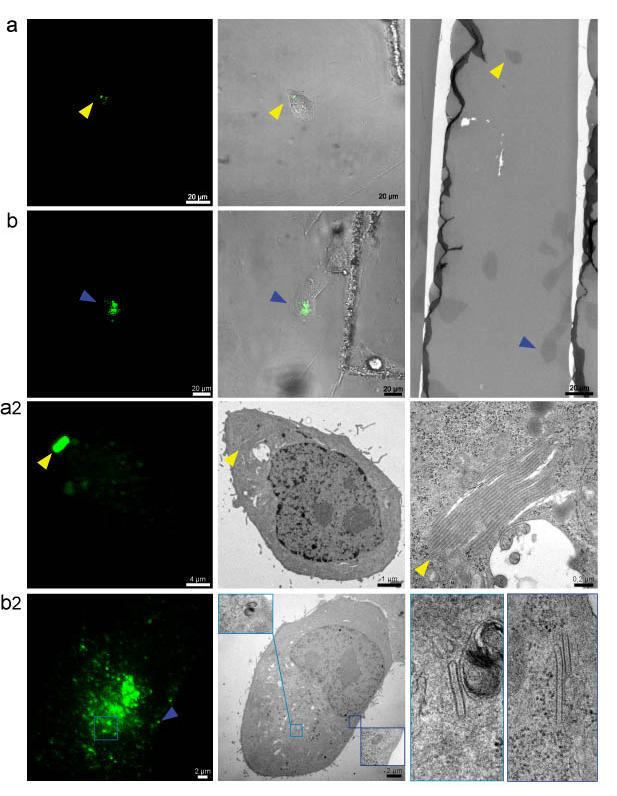

Supplement: Figure S3 — CLEM analysis of cells expressing mYFP-Lang. M10 cells expressing mYFP-Lang were processed for CLEM as in Fig. 2 . On the same Aclar® culture support, two cells with different phenotypes were observed: the first (a, a2, yellow arrowhead) displayed small puncta which were identified ultrastructurally as BG-like OSER; the second (b, b2, blue arrowhead) displayed classical, pericentriolar rod-shaped BGs. (TIF) [file pone.0060813.s003.tif]

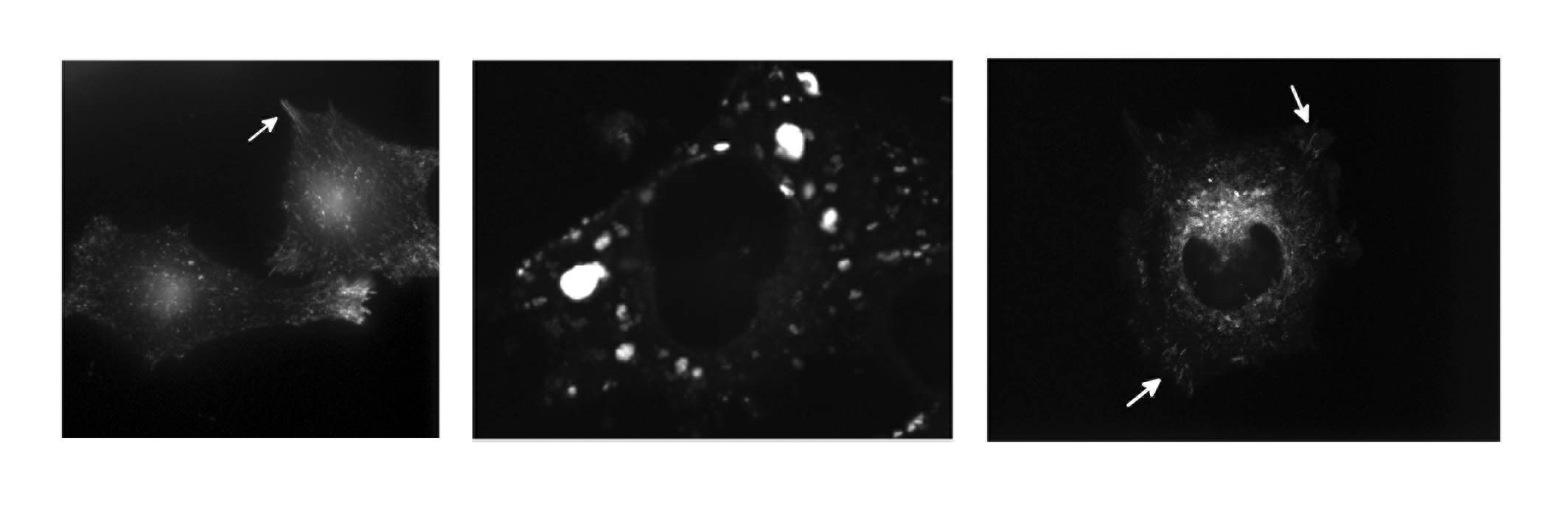

Supplement: Figure S4 — Restoration of the mobility of YFP-Lang with the A206K monomerizing mutant of YFP. Maximum intensity projections, generated from t-stacks of images acquired during FRAP experiments, are depicted for M10-Lang-YFP (left panel), M10-YFP-Lang (middle panel) and M10-mYFP-Lang (right panel) cells. The mobility of the Langerin/YFP chimeras can be roughly estimated from the presence of elongated, linear structures corresponding to small vesicles in motion, particularly visible in the immediate proximity of the plasma membrane or in the pericentriolar region (arrows). These elongated structures are nearly absent in M10-YFP-Lang cells, but similarly present in M10-Lang-YFP and M10-mYFP-Lang cells. (TIF) [file pone.0060813.s004.tif]

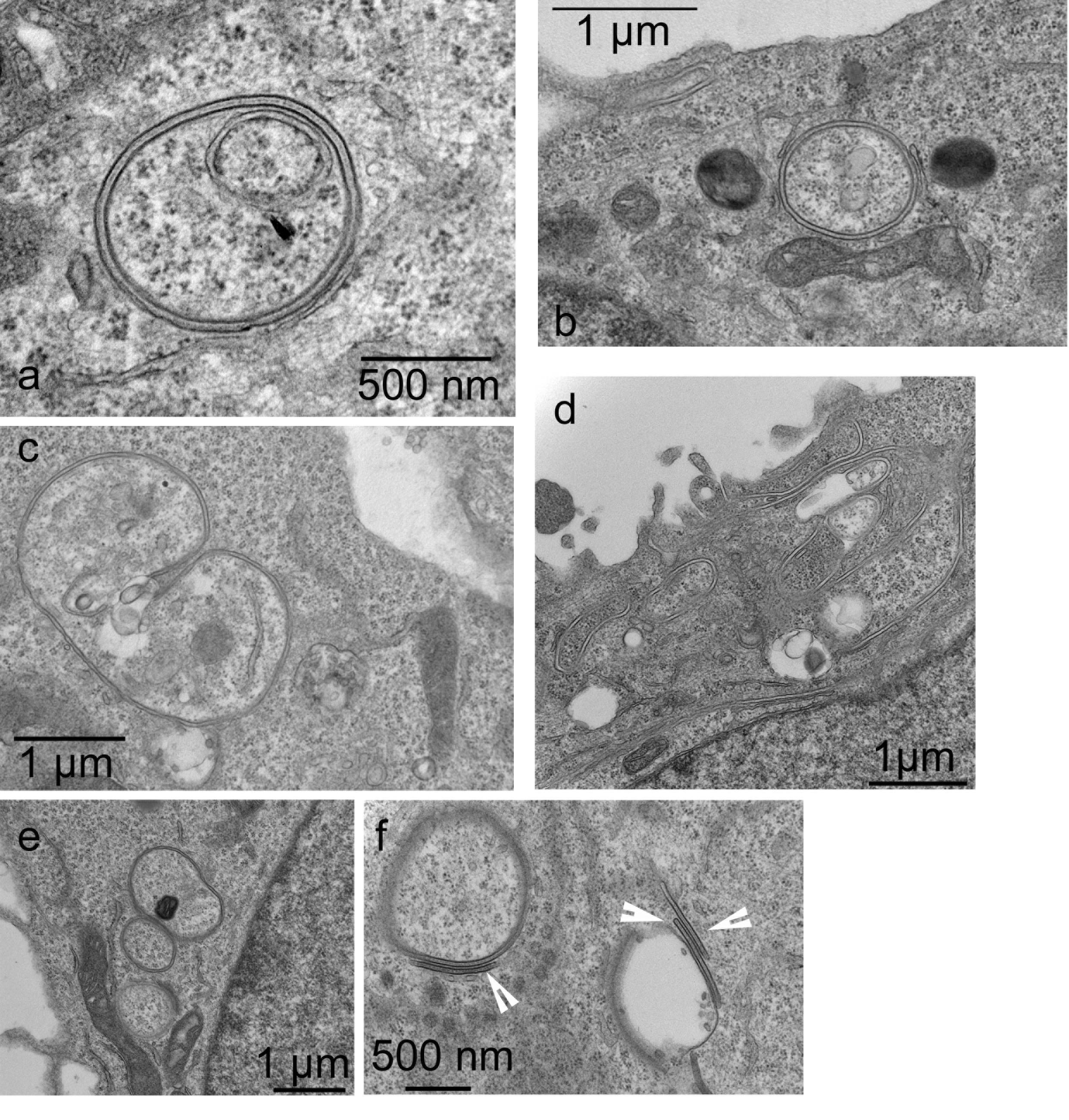

Supplement: Figure S5 — M10 transfected cells expressing YFP-LangE293A were fixed included in Epon. Sometimes, the central striation characteristical to classical BGs were noticed (white arrows). (TIF) [file pone.0060813.s005.tif]

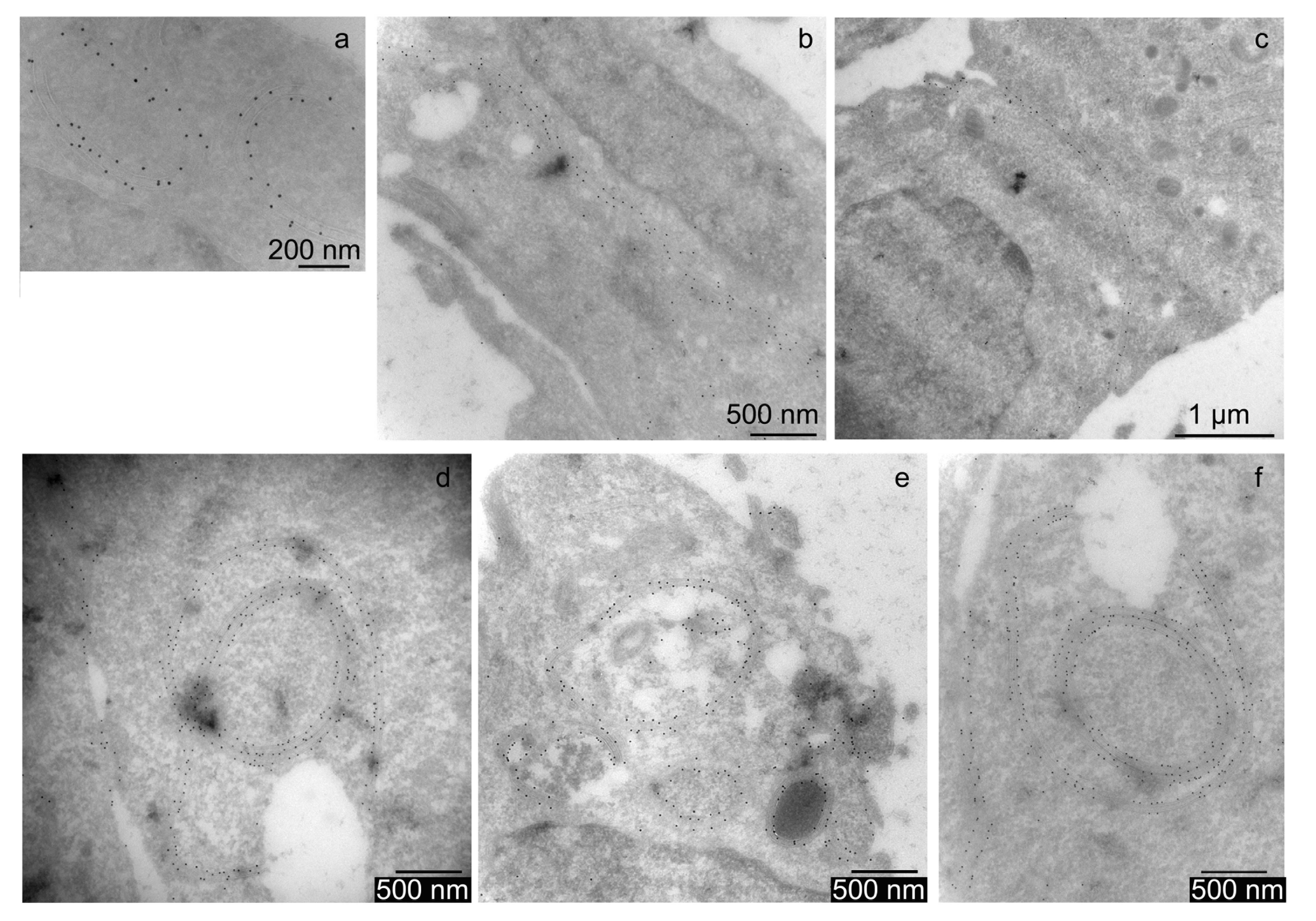

Supplement: Figure S6 — M10 transfected cells expressing YFP-LangE293A were fixed with 0.2% gluteraldehyde 2% paraformaldehyde, frozen in liquid N2, cryosections were labeled with rabbit polyclonal anti-GFP Abs, revealed with protein A conjugated 10 nM gold particles (PAG, Utrecht) and analyzed on CM120 electronic microscope (FEI). (TIF) [file pone.0060813.s006.tif]
